# Long-Term Quality of Life after COVID-19 Infection: Cross-Sectional Study of Health Care Workers

**DOI:** 10.3390/ijerph21020235

**Published:** 2024-02-17

**Authors:** Moussa Antar, Hansjoerg Ullerich, Andreas Zaruchas, Torsten Meier, Ricarda Diller, Ulrich Pannewick, Sameer A. Dhayat

**Affiliations:** 1 Department of Internal Medicine, Gastroenterology, and Pneumology, Brüderkrankenhaus St. Josef Paderborn, Husener Straße 46, 33098 Paderborn, Germany; m.antar@bk-paderborn.de (M.A.); a.zaruchas@bk-paderborn.de (A.Z.); u.pannewick@bk-paderborn.de (U.P.); 2 Department of Medicine, Gastroenterology, and Infectiology, University Hospital Muenster, Albert-Schweitzer-Campus 1 (A14), 48149 Muenster, Germany; hansjoerg.ullerich@ukmuenster.de; 3 Department of Anaesthesiology, Operative Intensive Care Medicine, and Pain Therapy, Brüderkrankenhaus St. Josef Paderborn, Husener Straße 46, 33098 Paderborn, Germany; t.meier@bk-paderborn.de; 4 Department of General and Visceral Surgery, Brüderkrankenhaus St. Josef Paderborn, Husener Straße 46, 33098 Paderborn, Germany; r.diller@bk-paderborn.de

**Keywords:** long-term quality of life (QoL), COVID-19 pandemic, health care workers, post-COVID syndrome, WHOQOL-BREF

## Abstract

(1) Background: Post-COVID syndrome is defined as symptoms that occur simultaneously with or after a COVID-19 infection, last for 12 weeks, and are not due to another diagnosis. Limited data are available on people’s long-term quality of life following a COVID-19 infection. The aim of this cross-sectional study was to investigate the long-term quality of life after COVID-19 among employees of a hospital in Germany and to identify risk factors. (2) Methods: A monocentric, cross-sectional study was conducted using the validated and digitized WHOQOL-BREF questionnaire via Netigate® between 10/2022 and 02/2023. Data on the quality of life and global health status were collected in the following four domains: physical health, mental health, social relationships, and the environment. (3) Results: The response rate was 73.8 % (923/1250). Furthermore, 63.4 % of the hospital staff respondents reported at least one persistent symptom after a COVID-19 infection, leading to significant differences in quality of life. Pre-existing conditions, persistent symptoms, and disabilities after a COVID-19 infection as well as a high BMI, no partnership, and a low educational level were found to significantly contribute to a low long-term quality of life. (4) Conclusions: Obesity, a lack of partnership, and a low level of education were independent risk factors for a lower quality of life post-COVID-19 infection in this cohort of hospital staff. Further multicenter studies are required to validate the incidence and their suitability as independent risk factors for post-COVID syndrome.

## 1. Introduction

The SARS-CoV-2 pandemic broke out in late 2019 at a food market (Huanan South China Seafood Market) in Wuhan, Hubei Province, China, and has claimed millions of lives worldwide. Current knowledge suggests that SARS-CoV-2 crossed the species barrier from bats to humans, with no intermediate host identified to date [1,2,3]. By the early 2020s, it was clear that this novel viral disease would become an ongoing global crisis. A public health emergency was declared in more than 200 countries worldwide. The World Health Organization (WHO) classified Coronavirus Disease 2019 (COVID-19) as a pandemic on 11 March 2020 [4,5,6,7]. According to the WHO Coronavirus dashboard [8], which shows the number of cases in the COVID-19 pandemic, by 27 July 2023, there had been a total of 768,560,727 confirmed COVID-19 cases worldwide, of which 6,952,522 had resulted in deaths. On 5 May 2023, three years after the start of the pandemic, the WHO lifted the global COVID-19 emergency.

**Figure 1:**
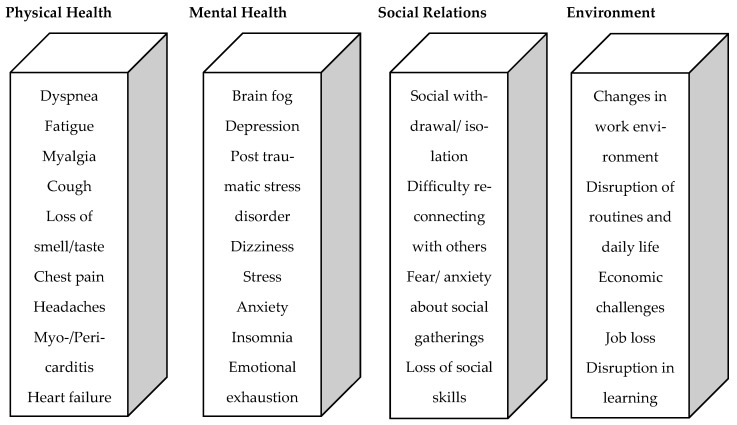
Visualization of the effects of long-/post-COVID syndrome on the domains of quality of life.

Following a COVID-19 infection, a person may experience persistent or new symptoms for weeks afterwards. Long COVID, also known as post-COVID, is a complex syndrome that can include a wide range of symptoms. To date, more than 200 different symptoms have been associated with long-/post-COVID. These nonspecific symptoms can affect different organ systems and include physical, neurological, and psychological complaints [9]. These complaints include general fatigue, myalgias, persistent shortness of breath, loss of smell and taste (anosmia), insomnia, chest pain, headache, arthralgia, cough, diarrhea, and psychological problems [10,11,12,13,14,15,16]. Whether the person had a mild, moderate, or acute COVID-19 course in the acute phase of the infection does not matter. The persistent limitations can be divided into two phases [11,17,18]: during the period of 4 to 12 weeks, it is called subacute COVID-19 syndrome, while after 12 weeks, it is referred to as chronic or post-COVID syndrome [19]. The impact of long-/post-COVID syndrome on quality-of-life domains is visualized in Figure 1. 

To make a final diagnosis of long-/post-COVID syndrome, people infected with COVID-19 should fall into one of the following three categories: persistent symptoms, the development of new infection-associated symptoms as a secondary condition, or a worsening of pre-existing underlying disease(s) [20]. Current knowledge suggests that up to 10–20% of COVID-19-infected individuals will develop long-/post-COVID syndrome, regardless of whether the person had a mild, moderate, or acute COVID-19 course in the acute phase of the infection [18,19]. One can assume that affected long-/post-COVID patients are and may remain limited in their activity, participation, and quality of life (QoL) [12].

The female gender, age 40 years or younger, the presence of an underlying mental or physical illness (e.g., asthma, diabetes mellitus, hypertension, and severe acute COVID-19 symptoms), financial hardship or losses, and unemployment are considered risk factors for increased psychological distress in the context of the COVID-19 pandemic [21,22,23,24]. Meta-analyses have revealed that post-COVID syndrome is associated with fatigue, brain fog, and depression, resulting in a reduced QoL and an inability to return to work. Furthermore, individuals with pre-existing psychological distress, including anxiety, loneliness, and depression, are at an elevated risk of having a severe COVID-19 course as well as developing long COVID [25,26,27,28]. There are other risk factors, among which employment in the healthcare sector, being overweight, and obesity stand out [14,21]. In addition to the risk factors already identified for a low quality of life, negative changes in sleep quality (insomnia, difficulty falling asleep and staying asleep) and dietary behaviors and alcohol, tobacco, and drug consumption were also identified, which may be due to the particularly high burden on healthcare staff caused by the COVID-19 pandemic [29,30,31,32]. 

Several studies have demonstrated that up to 74.1 % of healthcare workers infected with SARS-CoV-2 develop long-/post-COVID syndrome. The most common symptoms are fatigue/exhaustion, sleep disturbance, dyspnea/shortness of breath, cough, concentration/memory problems, headache, and a loss of taste/smell. The prevalence of post-traumatic stress disorder was also increased in healthcare workers, especially in those with severe COVID-19. The decrease in health-related QoL (HRQoL) after COVID-19 has been found to be correlated with the persistence of symptoms [13,33,34]. Furthermore, several studies on healthcare providers have revealed that levels of burnout increased significantly with an excessive workload and a fear of COVID infection and correlated negatively with QoL [35,36,37,38].

HRQoL is a significant predictor of overall health and well-being and is, therefore, highly relevant to public health. The COVID-19 pandemic has had a negative impact on people’s physical, psychological, and social functioning, as well as an economic impact on society [5,16,21,22]. In the recently published literature, the Depression Anxiety and Stress Scale 21 (DASS21), the EuroQol-5D scale (EQ-5D), the 12-item Short Form Health Survey (SF-12), and the Nordic Musculoskeletal Questionnaire (NMQ) have been used to measure QoL after COVID-19 [35,36,37,38]. According to the WHO’s categorization, QoL can be divided into the following four domains: mental health, physical health, social relationships, and environmental QoL [39]. First, the mental health domain indicates whether a person is able to experience positive feelings and whether negative feelings are not currently predominant [21]. Second, the physical health domain refers to the ability to cope with daily life without pain, physical discomfort, or sleep disturbance [23]. Third, the social relationships domain indicates whether intact social relationships with other people exist, and whether mutual help can be expected [7,39]. Lastly, the environmental QoL domain indicates how safe a person feels in their environment, how satisfied they are with their financial situation, whether they have access to necessary information, and whether access to medical care is guaranteed [12,16].

COVID-19 infection leads to a variety of symptoms in the acute phase. In addition, the symptoms persist over time. As a result, QoL can be severely impaired in different areas of life (Figure 1). The predictors of persistent COVID-19 symptoms cannot be determined with certainty based on the current state of research and the aforementioned literature. Therefore, this study aimed to determine the incidence and risk stratification of a reduced QoL in the context of the COVID-19 pandemic among employees of a hospital in Germany [40].

## 2. Materials and Methods

In this cohort study, health care workers of a hospital in Germany were recruited from all medical departments in the period from 17 October 2022 to 28 February 2023 (total recruitment *N* = 1250, 100%).

The inclusion criteria for employees were being aged at least 18 years old and having legal capacity. Information about this study, a consent form, and the survey form were digitally sent to all 1250 hospital employees; 9.2 % (*n* = 85) of the staff members actively refused to participate, 14.9 % (*n* = 187) were excluded from this study due to incomplete questionnaires, and 26.1 % (*n* = 327) of the staff members did not respond to the e-mail.

To recruit more staff members for this COVID-19 study, a total of three reminder e-mails were sent out through the internal e-mail distribution list “PBalle” during the aforementioned recruitment period, along with calls for participation over the intranet for staff members without their e-mail accounts. Additionally, employees were actively and personally approached during working hours, and notices were placed in the wards and frequently visited corridors. A total of *n* = 651 (70.5 %) staff members were recruited through these measures.

Furthermore, a literature search on the topics of long COVID and post-COVID was conducted in PubMed (U.S. National Library of Medicine®, Bethesda, MD, USA), and corresponding guidelines on the topic were also searched for.

The study participants were given the validated standardized WHO Quality of Life [41] (WHOQOL; abridged version from 1998) questionnaire for COVID-19. This questionnaire was used to assess subjective QoL using a 5-point Likert scale. It measured the effects of the illness and consecutive impairments in everyday life or behavior, perceived subjective health, and functional status in the context of the respective culture, environment, and social status. This particular questionnaire was selected because it met the criteria of applicability, reproducibility, validity, and sensitivity. The 26 items of the WHOQOL-BREF COVID-19 questionnaire were prefaced with sociodemographic data (height, weight, sex, and age), highest educational level, marital status, the presence and number of children in the household, vaccination status with the number of vaccinations and vaccine manufacturer, and previous COVID-19 infections. The dichotomization of the variables of marital status and educational level was performed as follows: Employees could report their marital status as “living alone,” “living separately,” “divorced,” “widowed,” “living with a partner,” or “married.” For the statistical analysis, the first four marital statuses were defined as living without a partner, while “living with a partner” and “married” were considered living in a partnership. Employees could state their educational level as “no qualification,” “lower secondary school,” “intermediate school-leaving certificate,” “specialized school-leaving certificate,” “high school diploma,” or “university.” For the statistical evaluation, the first four degrees mentioned were defined as a low/medium level of education, while “high school diploma” and “university” were considered higher levels of education.

In the case of a previous COVID-19 infection, participants were also asked about persistent symptoms indicative of long-/post-COVID syndrome. The study participants were provided with a list of possible symptoms based on the Long-COVID Syndrome Patient Questionnaire from Median Clinics [42]. The severity of a COVID-19 infection was not recorded. Participants without a COVID-19 infection were asked about existing complaints unrelated to COVID-19. Additionally, questions were posed regarding possible nicotine/alcohol consumption and the presence of comorbidities (arterial hypertension, diabetes mellitus, bronchial asthma, lung diseases [COPD], and depression) before and after a COVID-19 infection or without one, along with the presence of occupational/employment disabilities for all study participants.

The 26 items of the WHOQOL-BREF COVID-19 questionnaire covered the dimensions of physical health, mental health, social relationships, and the environment. Five answer options were available, each of which could be assigned a point value from 0 to 4. A score of 0 indicated the greatest possible impairment of QoL, while a score of 4 indicated no impairment. The converted maximum score of 100 points signified no impairment of QoL, whereas a score of 0 indicated the maximum impairment. The domains of physical health, mental health, social relationships, and the environment were considered separately. At the end of the questionnaire, respondents were asked questions about employment disabilities, assistance, and the duration required to complete the questionnaire.

### Statistical Analysis

The results of this study were obtained through statistical analyses using IBM SPSS Statistics, version 28.0.1.1 (14) (SPSS Statistics for Windows, Armonk, NY: IBM Corp, USA) and Microsoft Excel 2019 for Windows. Graphical representations were created using SPSS, Microsoft Excel, and Microsoft Office Word 2019. Categorical variables are presented as absolute and relative frequencies, with graphical representations using bar charts. For continuous variables, histograms were used for graphical representations. Additionally, the median (M) and interquartile range (IQR) were considered for the continuous variables with a non-normal distribution, while the mean (M) and standard deviation were used for those with a normal distribution. Finally, the WHOQOL-BREF COVID-19 questionnaire was analyzed regarding the four dimensions among employees with and without a COVID-19 infection. The Mann–Whitney U test was employed for non-normally distributed primary and secondary questions, and a bivariate Spearman correlation analysis was used for independent and continuous samples of age, in years, and body mass index (BMI), in kg/m^2^, correlated to the respective converted score of the four domains, to compare employees with and without a COVID-19 infection. The *p* values were two-sided. For significance in the Mann–Whitney U test, the effect size (r) was calculated (Z/√ sample size N). Finally, a multiple linear regression analysis was conducted with continuous variables (age, in years, and BMI, in kg/m^2^), categorical variables (education and marital status) and binary variables (vaccinations, children in the household, and gender). The results are presented as regression coefficients. For the regression coefficients, the 95% confidence interval (CI), lower limit (UG), and upper limit (OG) were considered.

## 3. Results

**Figure 2:**
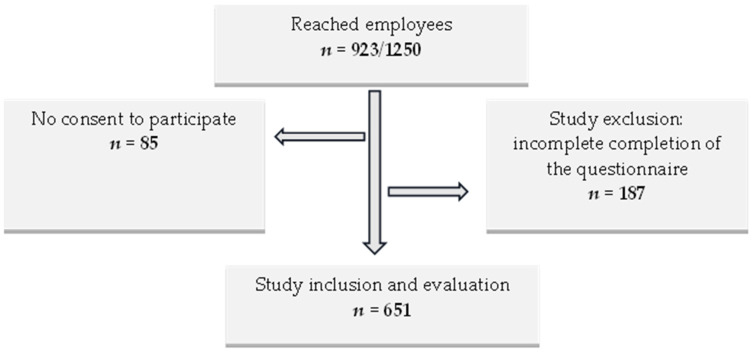
Flowchart of recruitment, from October 2022 to February 2023.

A total of 923 employees were reached from the initial population of 1250 employees at a hospital in Germany (Figure 2), for a response rate of 73.8 %. Furthermore, 85 employees (9.2 %) did not provide their consent to participate in this cross-sectional study, while 187 employees were excluded from this study due to incomplete questionnaires. Consequently, 651 employees (70.5 %) were included in the final evaluation. Figure 2 presents a flowchart of the recruitment process from October 2022 to February 2023.

### 3.1. Primary Outcome: Impact of COVID-19 Infection on QoL

**Figure 3:**
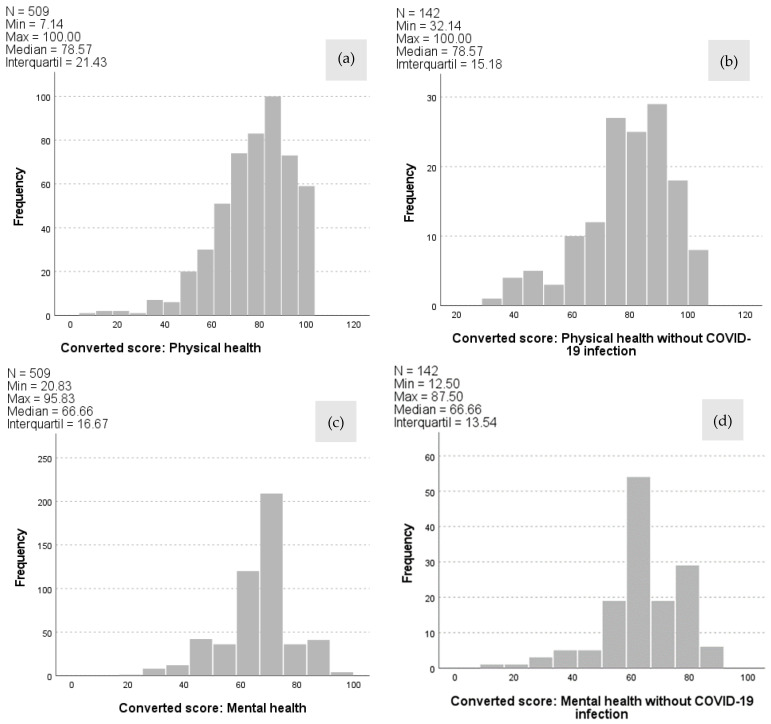

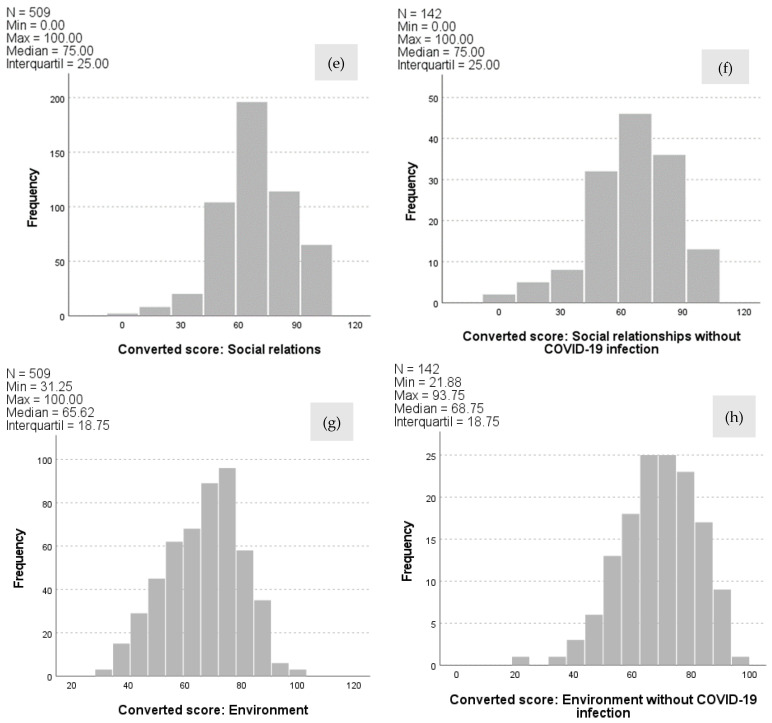
The WHOQOL-BREF questionnaire scores of COVID-19 affected and unaffected employees for the four domains of quality of life—namely, physical health (**a**,**b**), mental health (**c**,**d**), social relationships (**e**,**f**), and the environment (**g**,**h**)—are shown. The number of employees (N) and the minimum (Min), maximum (Max), median, and interquartile range (IQR) are given.

In total, *n* = 453 (69.6 %) of the participants were female. The mean age of the entire collective was 40.7 ± 12.7 years. Additionally, 69.5 % lived in a partnership, 54.1 % had children in the household, and 47.4 % reported a high level of education. Furthermore, 78.2 % of the workers had already been infected with COVID-19 without hospital treatment, while 96.9 % had a positive vaccination status against COVID-19 (Table 1). The study participants achieved a median WHOQOL score of 78.5 (IQR 21.4) points in physical health, 66.6 (IQR 16.6) points in mental health, 75.0 (IQR 25.0) points in social relationships, and 65.6 (IQR 18.7) points in environmental health. There were no significant differences in QoL between employees with and without a COVID-19 infection for the domains of physical health (*p* = 0.482), mental health (*p* = 0.129), social relationships (*p* = 0.236), and the environment (*p* = 0.064) (see Table 2 and Figure 3).

### 3.2. Secondary Outcomes: Modifiable and Nonmodifiable Predictors and Risk Factors for QoL

Furthermore, 63.4 % of the staff reported persistent symptoms after a COVID-19 infection. Significant differences in the QoL of employees with and without persistent symptoms after an infection were found for the four domains as follows: physical health (*p* < 0.001), mental health (*p* = 0.019), social relationships (*p* < 0.001), and the environment (*p* = 0.004) (Table 2). The employees were also asked about persistent symptoms following a COVID-19 infection. The most common symptoms were fatigue, tiredness, and a lack of strength in 67.8 % of the cases, followed by problems climbing stairs and muscular strains in 52.6 %, a difficulty breathing or shortness of breath on exertion in 43.9 %, poor concentration in 41.7 %, and headaches in 34.6 % (see Supplementary Figure S1 and Table S1). Moreover, 3.3 % of the employees reported COVID-19-related occupational disabilities. A low BMI correlated with a high QoL in all four domains as follows: physical health (*p* < 0.001), mental health (*p* = 0.023), social relationships (*p* = 0.050), and the environment (*p* = 0.001). A higher age correlated with a high QoL in the environment domain (*p* < 0.001) (Table 1). A lower QoL in the physical health domain correlated with the presence of pre-existing conditions after a COVID-19 infection (*p* < 0.001; r = 0.276), persistent symptoms after a COVID-19 infection (*p* < 0.001; r = 0.446), existing employment disabilities after a COVID-19 infection (*p* < 0.001; r = 0.146), a low educational level (*p* = 0.004; r = 0.111), and a lack of partnership (*p* < 0.001; r = 0.136). For the mental health domain, a lower QoL was found in the presence of previous illnesses after a COVID-19 infection (*p* < 0.001; r = 0.175), persistent symptoms without and after a COVID-19 infection (*p* = 0.056; r = 0.160/*p* = 0.019; r = 0.103), and a lack of partnership (*p* = 0.005; r = 0.111). A lower QoL for the social relationship domain correlated with the presence of previous illnesses after a COVID-19 infection (*p* = 0.015; r = 0.108), persistent symptoms after a COVID-19 infection (*p* < 0.001; r = 0.155), and a lack of partnership (*p* < 0.001; r = 0.141). Furthermore, a lower QoL was found for the environment domain for persistent symptoms after a COVID-19 infection (*p* = 0.004; r = 0.126), nicotine use before/after a COVID-19 infection (*p* = 0.050; r = 0.086/*p* = 0.046; r = 0.088), a low educational level (*p* = 0.005; r = 0.109), and a lack of partnership (*p* < 0.001; r = 0.158) (Table 1 and Table 2). Furthermore, a high BMI proved to be a negative predictor, while living in a partnership was a positive predictor for the domain of physical health (*p* ≤ 0.001/*p* ≤ 0.001). Additionally, living in a partnership was a positive predictor for the domain of social relationships (*p* ≤ 0.001). Lastly, a higher age (*p* = 0.001), higher educational attainment (*p* = 0.032), and living in a partnership (*p* ≤ 0.001) were positive predictors, while a high BMI (*p* = 0.025) and children in the household (*p* = 0.002) were significantly negative predictors for the environment domain in this study cohort.

## 4. Discussion

### 4.1. Assessing Quality of Life in a German Hospital

The aim of this survey, among the staff of a hospital in Germany, was to determine the incidence and risk stratification of an impaired QoL in the context of the COVID-19 pandemic, with the goal of deriving a better care/treatment algorithm for those affected and preventing the resulting incapacity to work. QoL in the four domains of physical health, mental health, social relationships, and the environment was determined by various modifiable and nonmodifiable factors. This confirmed the secondary endpoints of this cross-sectional study and identified sets of characteristics that can be regarded as predictors of the development of a reduced QoL. The modifiable factors included BMI, educational level, partnership, and nicotine/alcohol consumption. The nonmodifiable factors included age, the presence of previous illnesses, persistent symptoms after/without a previous COVID-19 infection, and the presence of occupational disabilities after a previous COVID-19 infection. The primary endpoints of this cross-sectional study were the results of the WHOQOL-BREF questionnaire for the health domains of physical health, mental health, social relationships, and the environment in relation to a COVID-19 infection among hospital staff. As described in the literature, the COVID-19 pandemic, as a whole, has had a negative impact on people’s physical, mental, and social functioning [5,39].

There were no significant differences in the QoL between workers with and without a COVID-19 infection in the domains of physical health, mental health, social relationships, and their environment. Thus, a COVID-19 infection did not contribute significantly to the development of a lower QoL in the aforementioned four domains, even when workers reported having been infected with COVID-19 more than once. By contrast, Ghazy et al. demonstrated that health workers in the Arab world have a lower QoL in all four domains [43]. A COVID-19 infection had no significant effect on the QoL of staff in a hospital in Germany. Instead, several factors and secondary outcomes came together in this study and contributed to the development of a reduced QoL. Gillen et al. found that during the COVID-19 pandemic, healthcare workers’ well-being and QoL were significantly impaired due to the high stress from an increased workload and the fear of COVID-19 infection [44]. Constantly worrying about their own health and a possible infection had the potential to lead to insomnia, stress, and depression, which could, in turn, have a long-term negative impact on their QoL [34]. Noteworthily, chronic emotional stress can have a negative impact on the immune system and physical health [24].

### 4.2. Exploring Factors Influencing Quality of Life among Hospital Staff in Germany

Of the 651 survey participants at a hospital in Germany, the female gender was more strongly represented (*n* = 453; 69.6 % of participants) than the male gender (*n* = 192; 29.5 %). Thus, the female/male ratio was 2.36 to 1. The “diverse” gender, accounting for 0.5 % (*n* = 3), was not included in the statistical analysis due to the small number. The Mann–Whitney U test, applied to independent samples, indicated no significance for QoL in the four domains of physical health, mental health, social relationships, or the environment for both genders. This is in contrast to the Corona Health app study, where male participants had a higher mental and physical QoL than female participants. However, female participants were found to have a higher social QoL than male participants [39]. Furthermore, in contrast to the previous literature, the female gender was not found to be a risk factor for psychological vulnerability in our study population [17,33]. In other studies, female participants have exhibited higher prevalence rates of anxiety, depression, and stress compared with male participants, with a lower QoL [23,24]. Rashid et al. found that female health workers were more likely to have a lower mental and social QoL than male workers [45]. The effect of gender on QoL may depend on various factors, such as the cultural context or the population studied. It is also thought that biological, genetic, and hormonal differences affect the immune system and response. Other factors, such as social support, educational level, occupational status, and health history, also play a significant role [39].

The mean age of the participants in our study cohort was 40.7 ± 12.7 years. Among the female employees, the 18–35 age group (*n* = 169) was the most represented, accounting for 37.3 %. The 18–35 age group (*n* = 71) was also the most represented age group among the male staff, accounting for 36.9 %. A statistical analysis revealed that a higher age (*p* < 0.001) correlated with a high environmental QoL. This is consistent with another study that indicated that older people have a higher QoL than younger people [46]. Older individuals often possess more life experience and are better able to cope with life’s challenges, which can positively impact their QoL [45]. Additionally, older age was found to be a positive and significant predictor of environmental health (*p* = 0.001). Another study suggested that a good mental and environmental QoL may be associated with older age, leading to better mental health and a positive impact on one’s overall QoL [33].

Moreover, the mean BMI of female staff was 25.2 ± 5.2 kg/m^2^, while that of male staff was 26.5 ± 4.6 kg/m^2^. Significant results were obtained for the secondary endpoint of this cross-sectional study. A low BMI correlated with a high QoL in all four domains. Additionally, BMI, in kg/m^2^, was a significantly negative predictor for the physical health domain. Furthermore, a high BMI, in kg/m^2^, was a significantly negative predictor for the environmental health domain. Further studies demonstrated that a high BMI was associated with an increased risk of developing persistent symptoms after COVID-19 [14]. Moreover, obesity is associated with an increased risk of long-/post-COVID, which may be linked to a reduced QoL [39].

Furthermore, 43.8 % of the employees (*n* = 285) had at least one child living in the household, while 55.9 % of the employees (*n* = 364) had no children living in the household. There was no significance for QoL in the four domains. However, in the multiple linear regression analysis, the presence of children in the household was a significantly negative predictor for the environmental health domain. Eicher et al. were also able to demonstrate that the presence of children in the household was associated with a lower social QoL under COVID-19 conditions [46].

Regarding living in a partnership, significant results were found in all four domains. Living in a partnership was correlated with a high QoL in the domains of physical health (*p* < 0.001), mental health (*p* = 0.005), social relationships (*p* < 0.001), and the environment (*p* < 0.001). Thus, living in a relationship/partnership is a highly protective factor. Living in a partnership is also a positive and significant predictor of physical health, social relationships, and environment. Social relationships through family and friends may contribute to an improved QoL after a COVID-19 infection [15]. By contrast, single people may be at an increased risk of social isolation and loneliness during or after a COVID-19 infection, which could have a negative impact on their mental health [23,24]. The presence of strong social support, whether from family, friends, or other social networks, may improve one’s QoL [47]. Rashid et al. demonstrated that married health professionals tended to have a better physical QoL than their single counterparts [45].

In addition, 47.4 % of the employees (*n* = 309) reported a high educational level (university or high school diploma), while 52.3 % (*n* = 341) reported a lower/middle level in our study cohort. There were significant differences in QoL with a higher education in both the physical health domain (*p* = 0.004) and the environment domain (*p* = 0.005). Additionally, a higher level of education was a significantly positive predictor for the environmental health domain (*p* = 0.032). This is consistent with the Corona Health app study, which demonstrated that a higher level of education was associated with a higher QoL than a lower level of education [39]. In contrast to the study of Zurek et al. [7], Eicher et al. also found a positive correlation between higher education and a higher QoL. In general, people with a higher education have better access to resources and more job opportunities, which can contribute to a higher QoL [46].

Significant differences existed in the QoL in the presence of pre-existing conditions and a COVID-19 infection in the domains of physical health, mental health, and social relationships. Consistent with other studies, pre-existing conditions such as cardiovascular disease, diabetes, lung disease, or anxiety disorders increase people’s risk of developing persistent symptoms associated with a reduced QoL [33]. Studies have also suggested that pre-existing conditions increase the risk of developing post-COVID syndrome [5,18]. In our study, 28.8 % of the 142 workers (*n* = 41) who had not been infected with COVID-19 reported a pre-existing condition. In contrast to the employees who had been infected with COVID-19, no significant effects were found in the QoL in the four domains.

Moreover, 96.9 % of the staff (*n* = 631) in our study had a positive vaccination status. The most commonly used vaccine was Comirnaty® (BioNTech/Pfizer) for the first to fourth vaccination, while the second vaccine of choice was Spikevax® (Moderna) for all four vaccinations. Correlational analyses indicated no significant effect on the QoL in the four domains in all vaccination groups. This is consistent with recent studies that have demonstrated that limited data currently exist on the efficacy of COVID-19 vaccines in preventing long-/post-COVID [17,45]. It is estimated that approximately 15 % of unvaccinated patients may be affected by persistent symptoms and may thus experience a reduced QoL [17].

### 4.3. Differential Impact on Quality of Life

This study found significant differences in the QoL of employees with and without persistent symptoms after a COVID-19 infection in the physical health, mental health, social relationships, and environment domains. This is consistent with the meta-analysis study of Malik et al., who found that patients with persistent symptoms reported a low QoL [16]. Further studies investigated persistent symptoms in patients after a COVID-19 infection and found that 76 % of them still had at least one symptom after 186 days. Long-/post-COVID can affect people with both mild and severe symptoms. It can include more than 200 symptoms that may significantly impact individuals in various aspects of their daily life and their QoL [9,18]. Due to this complex clinical picture, a multidisciplinary approach to long-/post-COVID is required, involving different disciplines with a focus on symptom-oriented therapy to ultimately improve the QoL in all four domains [18].

Additionally, 30.2 % of the 142 employees (*n* = 43) without a COVID-19 infection also reported symptoms and complaints. Similar to COVID-19-infected workers, there were significant differences in the QoL between workers with and without symptoms, independent of a COVID-19 infection, in both the physical and mental health domains (*p* ≤ 0.001; *p* = 0.056). No significant results were found for social relationships and the environment. Due to the possible absence of a COVID-19 infection, social contacts were maintained despite the reported complaints; thus, the QoL was not significantly negatively affected in the domains of social relationships and the environment. In summary, several studies have also reported a reduced QoL in the presence of symptoms/complaints with and without a prior COVID-19 infection [13,33].

Furthermore, no significant differences were found in the QoL of workers with and without an occupational disability after a COVID-19 infection in the domains of mental health, social relationships, and the environment. Significant differences in QoL were found between workers with and without an occupational disability after a COVID-19 infection for the physical health domain (*p* < 0.001). This is partly consistent with the Corona Health app study, which reported a lower mental and physical QoL in nonworkers [39]. For workers who were not infected with COVID-19, there were no significant differences in any of the four domains.

### 4.4. Limitations

This cross-sectional study has some limitations. The QoL data were collected at the end of the pandemic. Additionally, it did not differentiate between the different COVID-19 mutations during the pandemic, nor did it classify the severity of COVID-19 infection in the sample. In this context, it did not consider whether a COVID-19 infection could potentially have further effects on one’s long-term QoL due to different mutations of the virus, as it is known that different COVID-19 mutations lead to different levels of infection severity. Furthermore, the timing and format of the interviews may have made people more or less aware of their experiences and symptoms. For example, they may have forgotten their COVID-19 symptoms or dramatized symptoms they experienced shortly after a COVID-19 infection. We used a validated self-report questionnaire to anonymously identify specific symptoms of global health and QoL. Despite the disadvantage of providing invalid answers, especially on sensitive questions, the respondents are much closer to the issues in anonymous self-report questionnaires and tend to be more accurate. The random selection of participants also enables the generalization of the findings. Moreover, using self-report questionnaires as screening instruments enables a large amount of quantitative data to be collected quickly without major administrative or financial effort [47]. The strength of this study lies in the interdisciplinary view of health care workers and the inclusion of different departments. This provides broader insights into the health and working conditions of health workers in times of the COVID-19 pandemic. By including different disciplines and departments, different experiences, pressures, and challenges could be identified. Doing so could also identify similarities and differences between different professional groups. This survey was conducted among health care workers from a single tertiary-care hospital in Germany. The cross-sectional study design is useful for monitoring and evaluating the prevalence of high-risk groups; however, it limits causal understanding. Although the statistical generalizability of our findings is limited, an alignment with recent research suggests good transferability [15,23,24,33,43,44,45,48,49,50,51].

Despite these limitations, this cross-sectional study plays a valuable role in the scientific discourse and understanding of the long-term QoL after a COVID-19 infection among hospital staff.

## 5. Conclusions

The present cross-sectional study of hospital staff in a hospital in Germany examined the influence of a COVID-19 infection on QoL in the areas of physical health, mental health, social relationships, and the environment. QoL was determined by various modifiable and nonmodifiable factors. A previous COVID-19 infection did not have a significant impact on QoL in any of these domains.

A higher BMI had a negative effect on physical health, while living in a partnership had a positive effect on physical health and social relationships. Regarding the environment, older age, higher education, and living with a partner had positive effects, while a higher BMI and having children in the household had negative effects on the environment. Overall, the results of this cross-sectional study provide crucial insights into how different factors are related to one’s QoL. It should be noted, however, that correlations and predictors do not automatically imply causation. Further multicenter studies are required to validate risk factors of post-COVID syndrome. The present results contribute to an enhanced understanding of QoL and its relationship with a COVID-19 infection and other factors, which may have important implications for the healthcare and the well-being of the population. There is currently no causal therapy. For persistent symptoms and complaints, therapy should be multidisciplinary and symptom oriented, and it should include components of lifestyle modifications with appropriate information on diet, exercise, and healthy lifestyles.

## Figures and Tables

**Table 1:** Descriptive statistics of all study participants.

Characteristics	All Employees	* Statistics *p*-Value, 1–4
Total, *n* (%)	651 (100)	
**Gender, *n* (%)**		1: 0.257
Female	453 (69.6)	2: 0.486
Male	192 (29.5)	3: 0.291
Other	6 (0.9)	4: 0.383
**Age (years)**		1: 0.881
Female	40.2 (±12.9)	2: 0.938
Male	41.7 (±12.9)	3: 0.462
Mean (SD)	40.7 (±12.7)	**4: <0.001**
**BMI (kg/m^2^)**		**1: < 0.001**
Female	25.2 (±5.2)	**2: 0.023**
Male	26.5 (±4.6)	**3: 0.050**
Mean (SD)	25.6 (±5.1)	**4: 0.001**
**Living in partnership, *n* (%)**		**1: <0.001**
Yes	453 (69.5)	**2: 0.005**
No	182 (27.9)	**3: <0.001**
no reply	16 (2.5)	**4: <0.001**
**Children in the household, *n* (%)**		1: 0.183
Yes	364 (55.9)	2: 0.672
No	285 (43.8)	3: 0.234
no reply	2 (0.3)	4: 0.195
**Educational level, *n* (%)**		**1: 0.004**
high	309 (47.5)	2: 0.163
low	341 (52.4)	3: 0.388
no reply	1 (0.2)	**4: 0.005**
**Vaccination Status, *n* (%)**		1: 0.139
Yes	631 (96.9)	2: 0.729
No	20 (3.1)	3: 0.468
		4: 0.800

* Shown are the *p*-values of the four domains of the WHOQOL-BREF questionnaire. IQR, Interquartile range; SD, standard deviation; 1: physical; 2: psychological; 3: social; 4: environment. The significant *p*-values are in bold type.

**Table 2:** Exploratory statistics of the study participants that were COVID-19 affected and unaffected.

Characteristics	COVID-19	COVID-19	* Statistics	
	Affected (A)	Unaffected (B)	*p*-Value, (1–4)	
**Total, *n* (%)**	509 (78.2)	142 (21.8)		
**Comorbidities, *n* (%)**			(A)	**1: <0.001**
Yes	110 (21.6)	41 (28.8)		**2: <0.001**
No	399 (78.3)	101 (71.1)		**3: 0.015**
				4: 0.110
			(B)	1: 0.600
				2: 0.860
				3: 0.956
				4: 0.961
**Nicotine consumption, *n* (%)**			(A)	1: 0.442
Yes	87 (17.0)	30 (21.1)		2: 0.744
No	422 (82.9)	112 (78.8)		3: 0.387
				**4: 0.046**
			(B)	1: 0.802
				2: 0.761
				3: 0.928
				4: 0.781
**Alcohol consumption, *n* (%)**			(A)	1: 0.239
Yes	118 (23.1)	38 (26.7)		2: 0.419
No	391 (76.8)	104 (73.2)		3: 0.499
				4: 0.103
			(B)	1: 0.070
				2: 0.816
				3: 0.083
				**4: 0.035**
**Symptoms and**			(A)	**1: <0.001**
**complaints, *n* (%)**				**2: 0.019**
Yes	323 (63.4)	43 (30.2)		**3: <0.001**
No	186 (36.5)	99 (69.7)		**4: 0.004**
			(B)	**1: <0.001**
				**2: 0.056**
				3: 0.168
				4: 0.196
**Occupational**			(A)	**1: <0.001**
**Disability, *n* (%)**				2: 0.216
Yes	17 (3.3)	15 (10.5)		3: 0.918
No	492 (96.6)	127 (89.4)		4: 0.979
			(B)	1: 0.490
				2: 0.857
				3: 0.210
				4: 0.699
**WHOQOL-BREF**				
**Median Score, (IQR)**				
physical health (a, b)	78.5 (21.4)	78.5 (15.1)		0.482
mental health (c, d)	66.6 (16.6)	66.6 (13.5)		0.129
social relations (e, f)	75.0 (25.0)	75.0 (25.0)		0.236
environment (g, h)	65.6 (18.7)	68.7 (18.7)		0.064

* Shown are the *p*-values of the four domains of the WHOQOL-BREF questionnaire; 1: physical; 2: psychological; 3: social; 4: environment; IQR, Interquartile range; SD, standard deviation; a, b, c, d, e, f, g, and h: refer to Figure 2. The significant *p*-values are in bold type.

## Data Availability

The data supporting the results of this study are available upon request and ethical approval from the authors. The data are not publicly available for privacy reasons.

## Supplementary Materials

The following supporting information can be downloaded at: https://www.mdpi.com/article/10.3390/ijerph21020235/s1: Figure S1 Persistent symptoms after COVID-19 infection Frequency per cent; Table S1 Frequency of symptoms following a COVID-19 infection.
