# Supplementary material for: Long-Term Quality of Life after COVID-19 Infection: Cross-Sectional Study of Health Care Workers
Source: Int J Environ Res Public Health. 2024 Feb 17;21(2):235. doi: 10.3390/ijerph21020235 (PMC10887886; doi:10.3390/ijerph21020235)
Supplement: Supplementary file 1 [file ijerph-21-00235-s001.zip › ijerph-2825440-supplementary.pdf]

Symptoms and complaints

Employees who have had a COVID-19 infection

**Persistent symptoms after COVID-19 infection**		**Frequency**	**per cent**
**Valid**	Yes	**323**	49.6
	No	186	28.6
	Total	509	78.2
**Missing**	without COVID-19 Infection	142	21.8
**Total**		651	100

Figure S1 Persistent symptoms after COVID-19 infection Frequency per cent

In 63.45% of the 509 employees (n = 323), symptoms persisted after a COVID-19 infection. Of these people, 71.82% (n = 232) were female, 27.55% (n = 89) were male and 0.61% (n = 2) were of mixed gender.

The following symptoms of the 323 employees affected were revealed in terms of number and percentage distribution. The three most common symptoms are highlighted in dark:

**Persistent symptoms after a COVID-19 infection**	**Frequency / per cent**
Increased tiredness, fatigue and weakness	**219 / 67,80**
Problems with climbing stairs and muscular strain	**170 / 52,63**
Problems breathing or the feeling of breathlessness on exertion	**142 / 43,96**
Loss of the ability to concentrate	135 / 41,79
Headache	112 / 34,67
persistent cough	94 / 29,10
Joint or muscle pain	92 / 28,48
Feeling of palpitations	87 / 26,93
Impaired ability to concentrate and difficulty formulating longer sentences	81 / 25,07

Word-finding disorder	80 / 24,76
Lack of drive and interest	78 / 24,14
Irritability	63 / 19,5
Problems with smelling or tasting	58 / 17,95
Anxiety and sleep disorders	47 / 14,55
Problems with balance and fine motor skills	42 / 13,00
Problems controlling feelings	35 / 10,83
Constipation or diarrhea	29 / 8,97
Loss of appetite	25 / 7,73
Ongoing preoccupation with experiences during the Covid-19 disease	22 / 6,81
Nausea and vomiting	9 / 2,78

Table S1 Frequency of symptoms following a COVID-19 infection
